# Dosimetric comparison of intensity‐modulated radiotherapy and volumetric‐modulated arc radiotherapy in patients with prostate cancer: a meta‐analysis

**DOI:** 10.1120/jacmp.v17i6.6464

**Published:** 2016-11-08

**Authors:** Wenting Ren, Chao Sun, Ningning Lu, Yingjie Xu, Fei Han, Yue Ping Liu, Jianrong Dai

**Affiliations:** ^1^ Department of Radiation Oncology, Cancer Hospital National Cancer Center Chinese Academy of Medical Sciences and Peking Union Medical College Beijing China; ^2^ Department of Nuclear Medicine Cancer Hospital, National Cancer Center Chinese Academy of Medical Sciences and Peking Union Medical College Beijing China

**Keywords:** IMRT, VMAT, prostate cancer, meta‐analysis

## Abstract

Intensity‐modulated radiotherapy (IMRT) and volumetric‐modulated arc therapy (VMAT) are two main radiotherapy techniques. The aim of this study is to explore which is the preferred technique in prostate treatment through the related publications and meta‐analysis. Two authors independently identified all relevant articles available regarding eligibility criteria on PubMed, Embase, and Cochrane Library databases until December 2015. Publication bias was evaluated with funnel plot, and statistical analyses were performed with Stata software. P<0.05 was thought statistically significant. Ten studies comprised a total of 110 patients; in total 110 IMRT plans and 110 VMAT plans that were included in this study. V40, V60, and V70 of rectum were significantly decreased in VMAT than in IMRT. However, V50 of rectum and V40, V50, V60, V70 of bladder had no statistical differences between IMRI and VMAT plans. Compared with IMRT, the treatment time and MUs of VMAT were significantly lower. VMAT protects rectum better than IMRT and improves the delivery efficiency. VMAT may be the preferred modality for treating prostate cancer.

PACS number(s): 87.55. D‐

## I. INTRODUCTION

Radiotherapy (RT) is widely used in the treatment of prostate cancer because it can greatly improve local regional control of tumors.^1^, ^2^ However, some toxic effects of adjacent non‐cancerous tissues, including gastrointestinal and genitourinary adverse effects, have been induced.^3^, ^4^ Thus, increasing the dose of planning target volume (PTV) while minimizing irradiation of surrounding healthy tissue becomes the main focus of developing RT techniques. Nowadays, RT techniques have emerged from two‐dimensional (2D CRT) techniques that based on X‐ray images and manual calculations to the three‐dimensional conformal radiotherapy (3D CRT) based on computerized tomography (CT) images incorporating computer algorithms. Intensity‐modulated radiotherapy (IMRT)^(5)^ as the development of 3D CRT becomes the most popular RT technique for its good sparing of surrounding critical organs and excellent conformity and homogeneity of PTV by using nonuniform radiation beam intensities and inverse planning method. With regard to both improving tumor control by increase prescription target dose and reducing genitourinary and gastrointestinal toxicity by reducing the volumes of the rectal and bladder walls exposed to high‐dose levels, IMRT becomes the standard technique to deliver external beam radiation therapy treatment to the prostate.^6^, ^7^ Among IMRT techniques, fixed field IMRT (shorten for IMRT) is the most common radiotherapy technique for its accurate output dose^8^, ^9^ and easy quality assurance.^(10)^

Volumetric‐modulated arc radiotherapy (VMAT) is the latest intensity‐modulated RT technique. Unlike fixed field IMRT, it simultaneously coordinates gantry rotation, MLC motion, and dose rate modulation by using a dynamic modulated arc, facilitating highly conformal treatment and optimal sparing of the critical structures near the target.^(11)^ What's more, VMAT has been proved to be the most efficient in monitor units (MUs) and treatment time compared with fixed field IMRT and tomotherapy^12^, ^13^ and has a wide range of applications in many kinds of tumor treatment especially for prostate treatment.^14^, ^15^, ^16^, ^17^ At present, a few comparative studies about IMRT and VMAT techniques have been established to explore which technique may be preferred for treating prostate cancer. From those studies, some discussions in sparing of organs‐at‐risk (OAR) and plan quality have emerged. Zhang et al.^(18)^ found that VMAT results in slightly better rectal sparing, while Cakir et al.^(19)^ found that VMAT and IMRT provide very similar OAR dose‐volume constraints. Quan et al.^(20)^ found that the irradiation of bladder can be significantly decreased in VMAT plan while Sale and Moloney^(21)^ found there is no significant differences between the VMAT and IMRT planning techniques, although the VMAT bladder doses are consistently lower than the IMRT plans. The inconsistent results in different studies may partly because of the small simple sizes and different planning methods, among many reasons.

With the aim of exploring which is the preferred radiotherapy technique in prostate treatment and resolving the inconsistencies which have arisen from previous studies, we conducted a systematic review using meta‐analysis to compare the sparing of OAR and treatment efficiency of IMRT and VMAT in prostate cancer, which may give some guidance for choosing technique in clinic.

## II. MATERIALS AND METHODS

### A. Search methods for identification of studies

We followed the recommendations of PRISMA statement,^(22)^ though our meta‐analysis was involved in observational studies. We searched the PubMed, Embase, and Cochrane library databases in English, without restrictions in years or publication status, by using the following keywords: “intensity modulated radiotherapy”, “IMRT”, “volumetric modulated arc radiotherapy”, “VMAT”, “Rapid arc”, “prostate”. These terms were combined with the search terms for the following study designs: ((prostate) AND (intensity modulated radiotherapy AND IMRT)) AND (volumetric modulated arc radiotherapy OR VMAT OR rapid arc). Then we used Google Scholar search engine and entered these keywords for a second collection of eligible studies. Review articles, editorials, case reports, congress abstracts, regarding recurrences and letters of opinion were excluded. The date of the last search was December 2015. Two independent reviewers selected and assessed the data using a standardized form for including and extracting data. When necessary, a group discussion was conducted to settle disagreements. Studies were included if they provided treatment time, MUs, dose‐volume histograms (DVHs) to rectum or bladder on IMRT and VMAT plans. The detailed exclusion strategies used were: 1) target volumes where whole pelvis or with simultaneous integrated boost, lymph node involvement; 2) VMAT with constant dose‐rate (cdrVMAT) delivery; 3) data without statistical analysis and standard deviation (SD).

### B. Data extraction and assessment of the risk of bias

The data from each included study were extracted including the first author, publication years, country, sample size, prescribed dose of IMRT and VMAT, treatment time, MUs, and average percent irradiated volumes of OAR (rectum and bladder) and standard deviation SD) at various radiation doses (from 40 Gy to 70 Gy, the interval of each level was 10 Gy) in dose‐volume histograms (DVHs).

With regard to the DVHs information, if the authors did not list the average percent irradiated volumes of rectum and bladder in table, Getdata Graph Digitizer software (http://www.getdata‐graph‐digitizer.com) was used to measure the data from the figures in the article. If the prescribed dose in the study was less than 70 Gy, the radiation dose of rectum and bladder were under 70 Gy, so we only extracted the data for radiation dose <70 Gy. If the article included both single‐arc VMAT and dual‐arc VMAT, we choose the dual‐arc results as it has been proved that dual‐arc is superior to single‐arc in terms of the compromise between plan quality and delivery efficiency.^(20)^

### C. Statistical analysis

Generic inverse variance method^(23)^ was used to calculate the standardized mean difference (SMD);^24^, ^25^ if I2 was less than 50%, p>0.1, there was no heterogeneity, a fixed effect model was used; otherwise, a random effect model was used.^24^, ^26^ The SMD of treatment time, MUs, irradiated volumes of rectum and bladder at various radiation dose (40 Gy, 50 Gy, 60 Gy, 70 Gy) for IMRT and VMAT treatment plans were compared. Publication bias was evaluated by funnel plot, and statistical analyses performed by Stata (version 12.1) software. P<0.05 was thought statistically significant.^(27)^

## III. RESULTS

### A. Study selection and characteristics of the included studies

**Figure 1:**
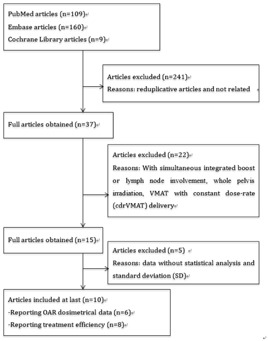
The flowchart of the retrieved studies and the main excluded reasons. OAR=organs−at−risk.

In total, 10 studies fulfilled the eligibility criteria. The flowchart of the retrieved studies and the main exclusion reasons are presented in Fig. 1. In these 10 studies, six report OAR dosimetric data (V40, V50, V60, V70 of rectum and bladder) and eight report treatment efficiency data (delivery time and MUs). The 10 studies comprised a total of 110 patients that generated 110 IMRT plans and 110 VMAT plans. The prescription dose was from 68 Gy to 78 Gy. The characteristics of the included studies are summarized in Table 1. Disagreement between the two independent reviewers occurred in one case and was consolidated after a group discussion.

**Table 1:** The characteristics of the included studies

*Study No*.	*Year*	*First Author*	*Country*	*Prescribed dose (Gy)*	*Sample Size*		*OAR (Level of the Dose) (Gy)*		*MUs*	*Delivery Time*
					*IMRT*	*VMAT*	*Rectum*	*Bladder*		
1	2009	Wolff D^(12)^	Germany	76	9	9	—	—	Yes	Yes
2	2011	Hardcastle N^(37)^	Australia	78	10	10	—	—	Yes	Yes
3	2011	Fogarty GB^(38)^	Australia	74/78	8	8	—	—	Yes	—
4	2011	Sale C^(21)^	Australia	75.6	8	8	V40,V50, V60,V70	V50,V60, V70	—	—
5	2011	Tsai CL^(13)^	Taiwan	78	12	12	V40	V40	Yes	Yes
6	2012	Quan EM^(20)^	USA	76	11	11	V40,V50, V60,V70	V40,V50, V60,V70	—	—
7	2012	Nguyen BT^(39)^	Australia	68	10	10	V50,V60	V60,V70	Yes	Yes
8	2013	Elith CA^(30)^	Australia	74	20	20	V40,V50, V60,V70	V40,V50, V60,V70	Yes	Yes
9	2014	Onal C^(34)^	Turkey	78	12	12	V40,V50, V60,V70	V40,V60, V70	Yes	—
10	2015	Cakir A^(19)^	Turkey	74	10	10	V50,V70	V50,V70	Yes	—

### B. Comparison of IMRT and VMAT

**Figure 2:**
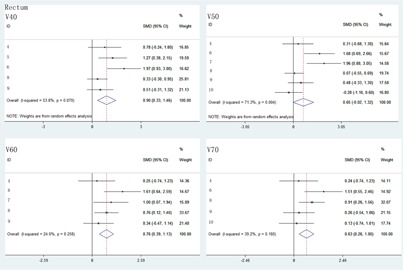
The comparison of radiation dose to rectum at V40, V50, V60, and V70 between IMRT and VMAT.

**Figure 3:**
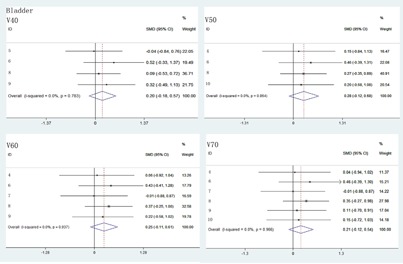
The comparison of radiation dose of bladder at V40, V50, V60, and V70 between IMRT and VMAT.

**Figure 4:**
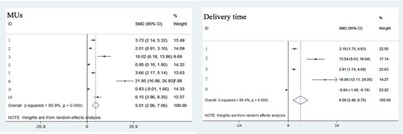
The comparison of MUs and delivery time between IMRT and VMAT.

For the radiation dose to rectum and bladder, V40, V60, and V70 of rectum were significantly decreased in VMAT than in IMRT (Fig. 2). However, V50 of rectum and V40, V50, V60, V70 of bladder had no statistical differences between IMRI and VMAT plans (Figs. 2 and 3). Compared with IMRT, the treatment time and MUs of VMAT were significantly lower (Fig. 4).

### C. Publication bias

**Figure A1:**
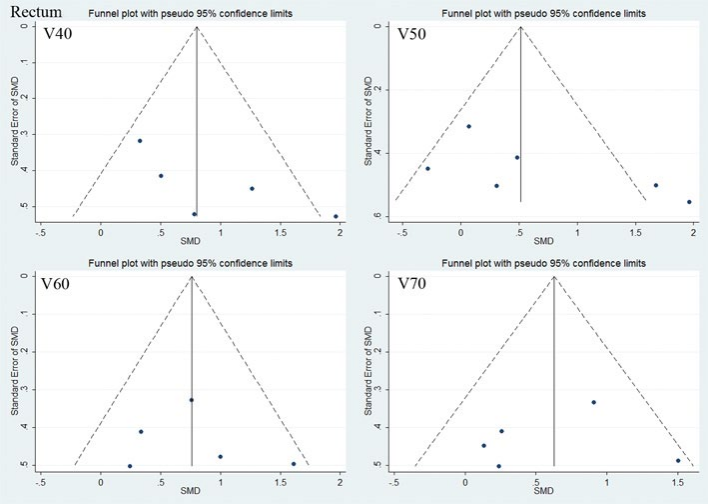
The funnel plots of rectum at radiation dose (40 Gy, 50 Gy, 60 Gy, 70 Gy).

**Figure A2:**
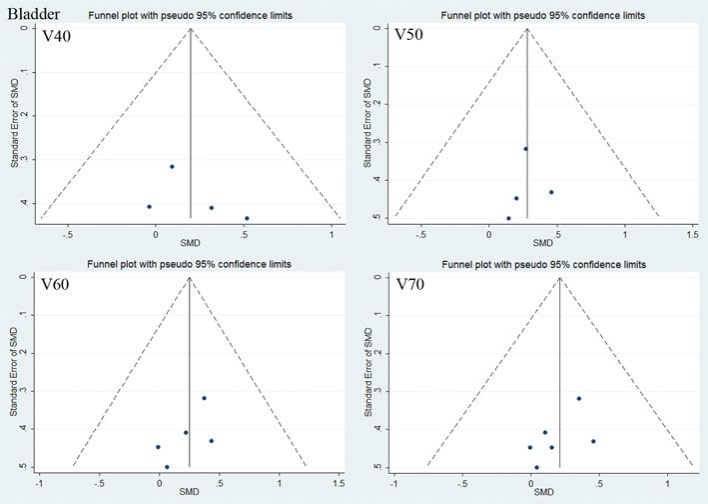
The funnel plots of bladder at radiation dose (40 Gy, 50 Gy, 60 Gy, 70 Gy).

**Figure A3:**
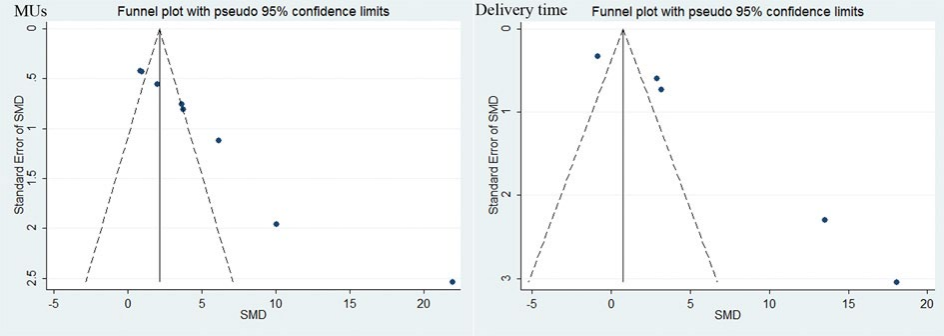
The funnel plots of MUs and delivery time.

The graphical Funnel plots revealed no obvious signs of publication bias for V40, V50, V60, 70 of rectum and bladder (see Figs. A1 and A2 in Appendix A). However, the funnel plots showed asymmetry of MUs and delivery time (Fig. A3). We also use Begger's test for assessing the publication bias and p<0.05 was considered statistically significant.^28^, ^29^ After Begger's test, the p‐value of MUs was found <0.05.

## IV. DISCUSSION

In this study, we detected the dosimetrical differences between IMRT and VMAT plans of prostate cancer using meta‐analysis. After combining multicenter results, our study showed that VMAT had significant advantages in reducing rectum dose and improving efficiency of treatment.

Rectum, which is extremely close to the PTV, is the most important organ at risk in prostate cancer. Sale and Moloney^(21)^ reported that the irradiated volume to rectum at dose of 40 Gy, 50 Gy, 60 Gy, and 70 Gy were significantly reduced in VMAT, compared to IMRT. However, the study by Elith et al.^(30)^ suggested that there was no significant reduction in average percent volumes irradiated by VMAT at doses of 40 Gy and 50 Gy. These heterogeneous results may be due to the small sample size, planning strategies, or optimization algorithm. After weighing the sample size of different studies, our meta‐analysis indicated that the average percent volumes of irradiated rectum (at doses of 40 Gy, 60 Gy, 70 Gy) were significantly lower in VMAT than in IMRT. There is no significant result in V50 which may be due to the limited number of included studies. However, the decrease of higher dose (40 Gy, 60 Gy, 70 Gy) was meaningful for clinical instructions since the rectal toxicity was relatively strong with the volume of the rectum receiving higher dose.^31^, ^32^ For the rectum irradiation dose lower than 40 Gy, our meta‐analysis has not been included because only two articles list V20 and V30 data.

Bladder is another important organ which should be protected in prostate cancer treatment. Quan et al.^(20)^ reported that VMAT plan performed better plan quality on bladder sparing than IMRT plan at dose of 30 Gy, 40 Gy, 50 Gy, 60 Gy, and 70 Gy. However, Sale and Moloney^(21)^ found that there was no significant reduction between VMAT and IMRT technique in bladder irradiated volume. After recalculation of the mean volumes of different dose levels from included studies, the meta‐analysis showed that there was no significant difference in protection of bladder between two techniques. This indicates to us that the radiation dose of bladder may be a result of comprehensive effect of planning methods such as algorithm, beam angles, and constraints, and not mainly decided by different radiotherapy techniques.

For the femoral heads and small bowel, no uniform and clear constraint had been described in the including studies, so we didn't compare femoral heads and small bowel in this meta‐analysis. A few studies had reported the radiation dose differences of femoral heads and small bowel in different techniques. Sale and Moloney^(21)^ and Palma et al.^(33)^ found that V30, V40, V45 of bilateral femoral heads were significantly lower in VMAT plans. For small bowel, the Sale study reported that there was no significant reduction in the volume of small bowel receiving lower doses in VMAT plans. Onal et al.^(34)^ also reported that there was no significant irradiation dose differences about D50 and Dmax between IMRT and VMAT in sigmoid colon.

For delivery efficiency, our meta‐analysis showed that MUs were significantly decreased in VMAT plans, which had been supported by numerous studies.^33^, ^35^, ^36^ The reason of significant reduction in MUs of VMAT came from the differences in the optimization algorithms used for VMAT and IMRT.^(20)^ However, Quan et al.^(20)^ reported that VMAT plans had higher MUs than IMRT plans, this may be due to their superior OAR sparing and they confirmed this by redoing the plan using loose constraints. As VMAT plans had fewer MUs, it was no doubt that VMAT delivery time was less than IMRT plans. Hardcastle et al.^(37)^ reported that about 29% per patient in‐room time would be reduced for VMAT technique, which was a significant gain in delivery efficiency, and would increase patient throughput accordingly.

There were some limitations in this study. First, the retrieved studies with which our meta‐analysis was carried out were observational. Although it is generally believed that findings from observational studies are not as accurate as those from randomized controlled trials, our meta‐analysis still gives some insight of the advantages between IMRT and VMAT technique after combining studies with small sample size. Furthermore, publication bias may exist in MUs and delivery time, the number and quality of retrieved studies may affect this. Additional prospective studies with large samples are essential in the future. Additionally, the toxicity of IMRT and VMAT with longer follow‐up is required for making clinical decisions.

## V. CONCLUSIONS

This study suggested that VMAT significantly reduced the irradiation volume of rectum at high‐dose level and improved the delivery efficiency. VMAT is a promising radiotherapy technique and may be considered as the first choice for prostate treatment.

## ACKNOWLEDGMENTS

This work was supported by National Natural Science Foundation (Grants 81402528).

## COPYRIGHT

This work is licensed under a Creative Commons Attribution 3.0 Unported License.

## APPENDICES

### Appendix A. Supplementary Materials
